# Supplementary material for: Quantification of Salmonella enterica serovar Typhimurium Population Dynamics in Murine Infection Using a Highly Diverse Barcoded Library
Source: bioRxiv. 2024 Jun 29:2024.06.28.601246. Preprint. [Version 1] doi: 10.1101/2024.06.28.601246 (PMC11230369; doi:10.1101/2024.06.28.601246)
Supplement: Supplementary file 1 [file media-1.pdf]

Supporting Information for

Quantification of *Salmonella enterica* serovar Typhimurium Population Dynamics and Bottlenecks in Murine Infection Using a Highly Diverse Barcoded Library

Julia A. Hotinger, Ian W. Campbell, Karthik Hullahalli, Akina Osaki, Matthew K. Waldor

Matthew K. Waldor
Email: mwaldor@bwh.harvard.edu

This PDF file includes:

Figures S1 to S8
Table S1
SI References

Other supporting materials for this manuscript include the following:

Dataset S1

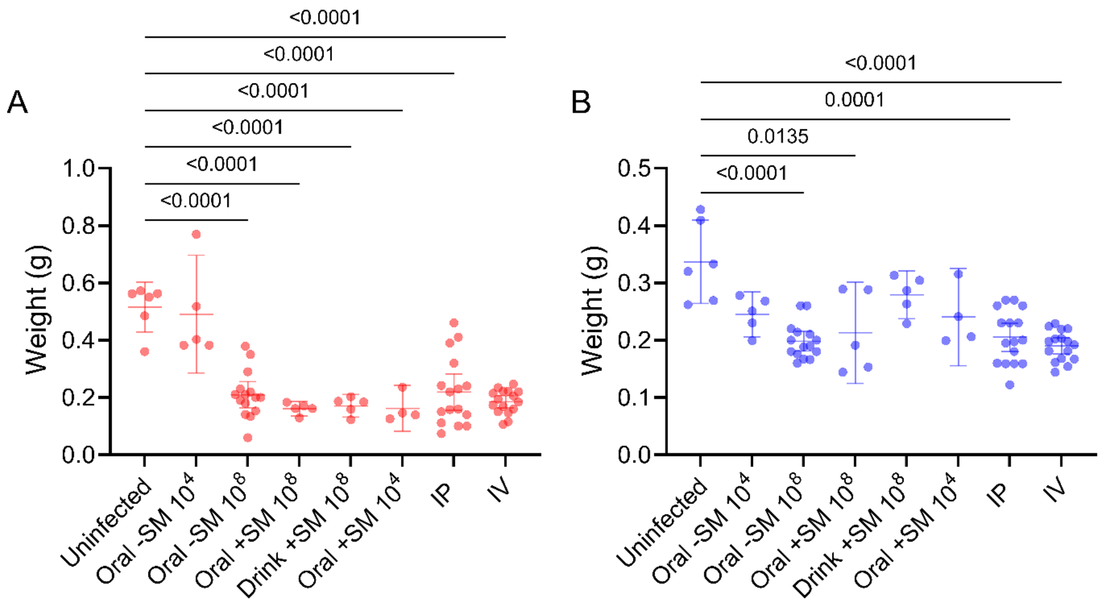

Fig. S1. Cecal (A) and colon (B) weights after different infection schemes. Mann-Whitney tests were used for statistical analyses.

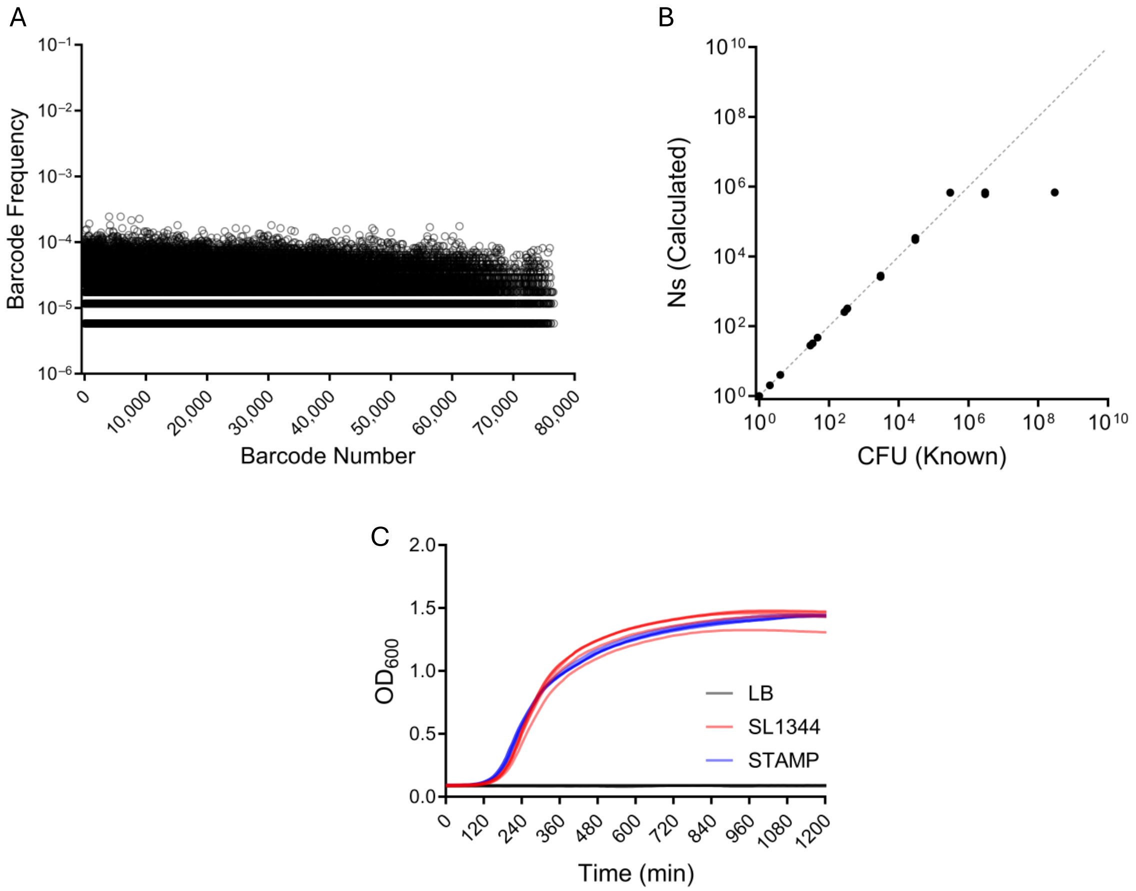

Fig. S2. S. Typhimurium barcoded library diversity and standard curve. A) Barcode frequency in the S. Typhimurium library shows a relatively even distribution of barcodes. **B)** Calibration curve indicating the library has a resolution limit of ~7x105. **C)** Growth of barcoded STAMP library in LB supplemented with SM is not different than the parent strain (SL1344).

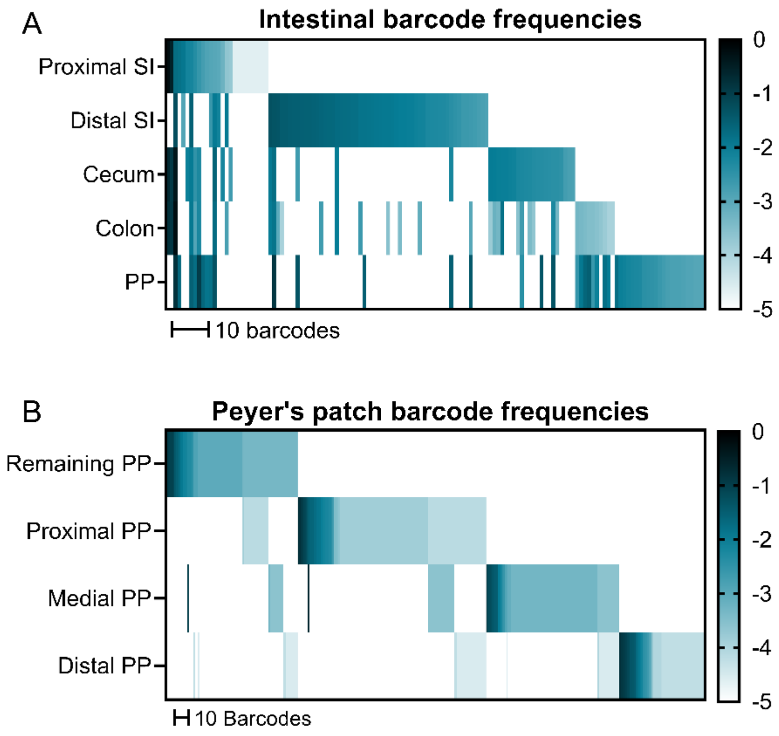

Fig. S3. Barcodes from different regions of the intestine and Peyer's patches from a single animal are largely distinct. A) Barcode frequencies of different regions of the intestine and **B)** individual Peyer's patches (PP) of an untreated mouse after orogastric gavage of *S*. Typhimurium. "Remaining PP" sample contains the PP from the animal not taken for individual analysis. Frequencies of barcodes are displayed as heatmaps (each box represents a barcode and darker colors represent increased frequency within the sample) and sorted by abundance in each sample sequentially.

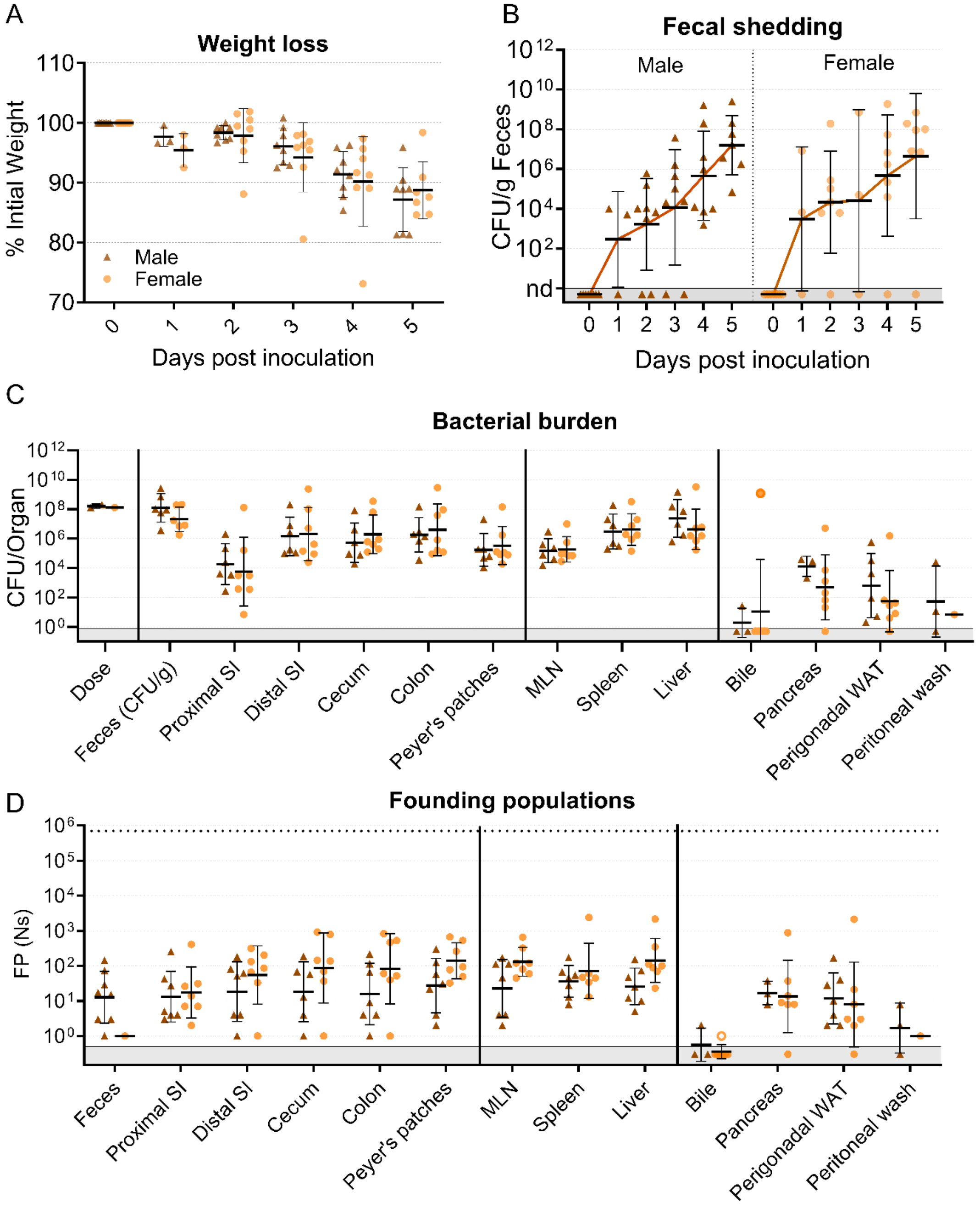

Fig. S4. Sex-disaggregated data in mice inoculated through orogastric gavage. A) Percentage of initial weight over time; means and standard deviations shown. **B)** Bacterial burden in feces. **C)** Bacterial burden in organs and fluids, Open circles indicate when the whole gallbladder was taken instead of bile. **D)** Founding populations (Ns) in organs and fluids. Geometric means and geometric standard deviations are used unless otherwise noted. -SM female n = 8, -SM male n = 8

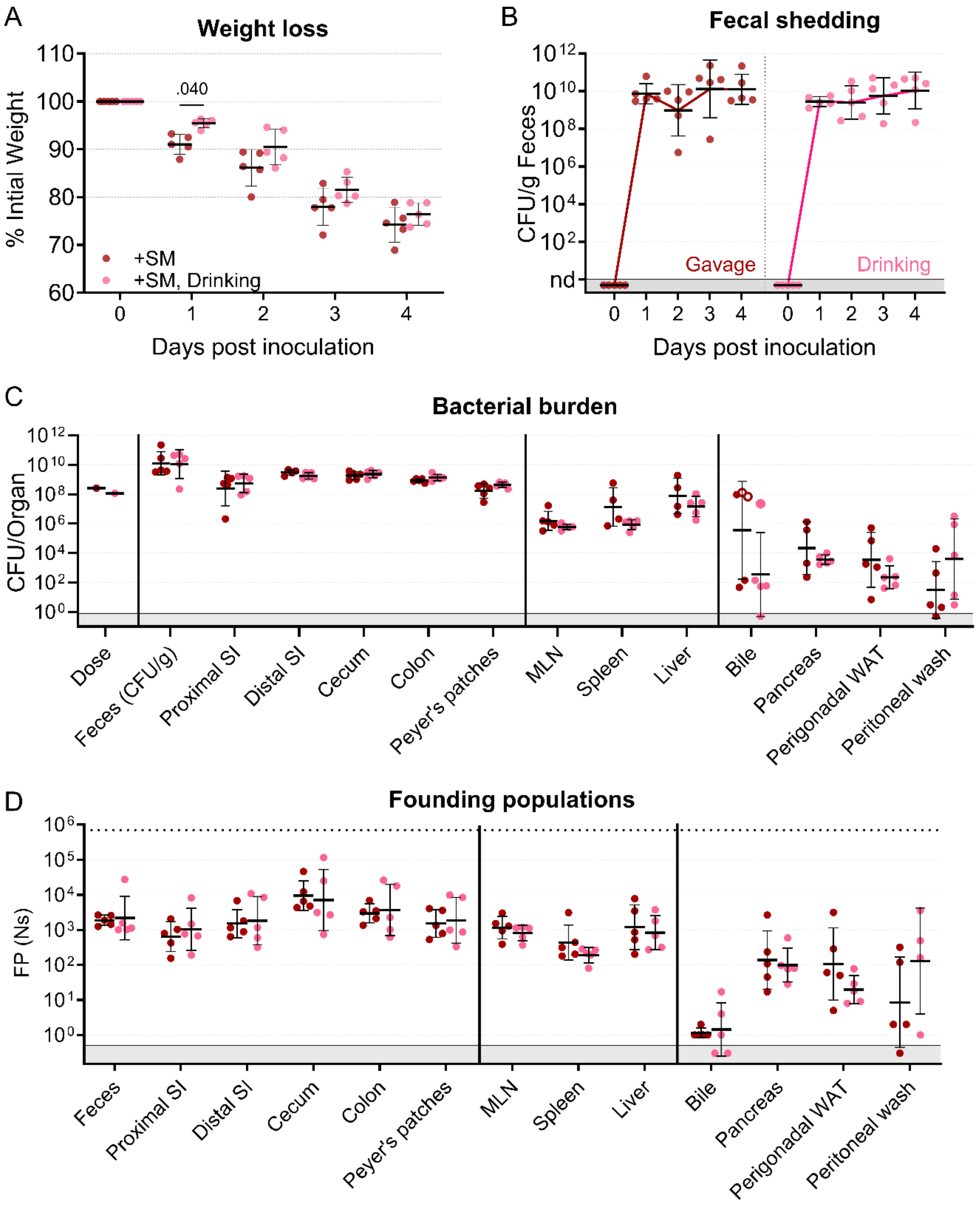

Fig. S5. Mice inoculated through orogastric gavage and drinking after streptomycin treatment. A) Percentage of initial weight over time; means and standard deviations shown. **B)** Bacterial burden in feces. **C)** Bacterial burden in organs and fluids, Open circles indicate when the whole gallbladder was taken instead of bile. **D)** Founding populations (Ns) in organs and fluids. Geometric means and geometric standard deviations are used unless otherwise noted. +SM female n = 5, and +SM, Drinking female n = 5. Mann-Whitney tests were used for statistical analyses. Values with $p < 0.1$ shown.

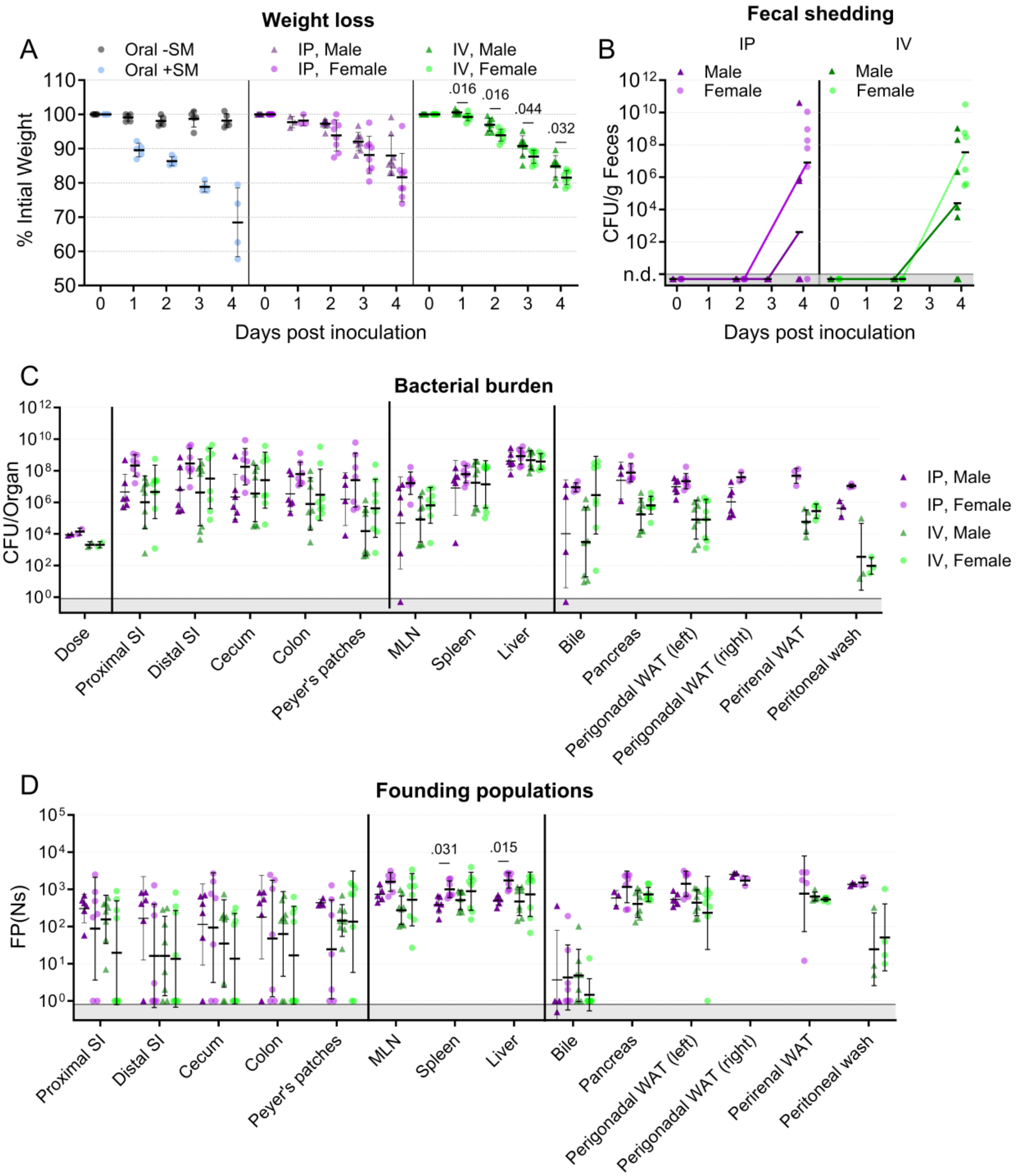

Fig. S6. Sex-disaggregated data in mice after inoculation with 10^3-10^4 CFU *S.* Typhimurium through different routes. A) Percentage of initial weight over time; means and standard deviations shown. **B)** Sex-disaggregated bacterial burden in feces. **C)** Bacterial burden in organs and fluids. **D)** Founding populations (Ns) in organs and fluids. Geometric means and geometric standard deviations are used unless otherwise noted. Oral -SM female n = 5, Oral +SM n = 5, IP male n = 8, IP female n = 8, IV male n = 8, and IV female n = 8. Statistical analysis is a Mann-Whitney test. Values with $p < 0.1$ are shown.

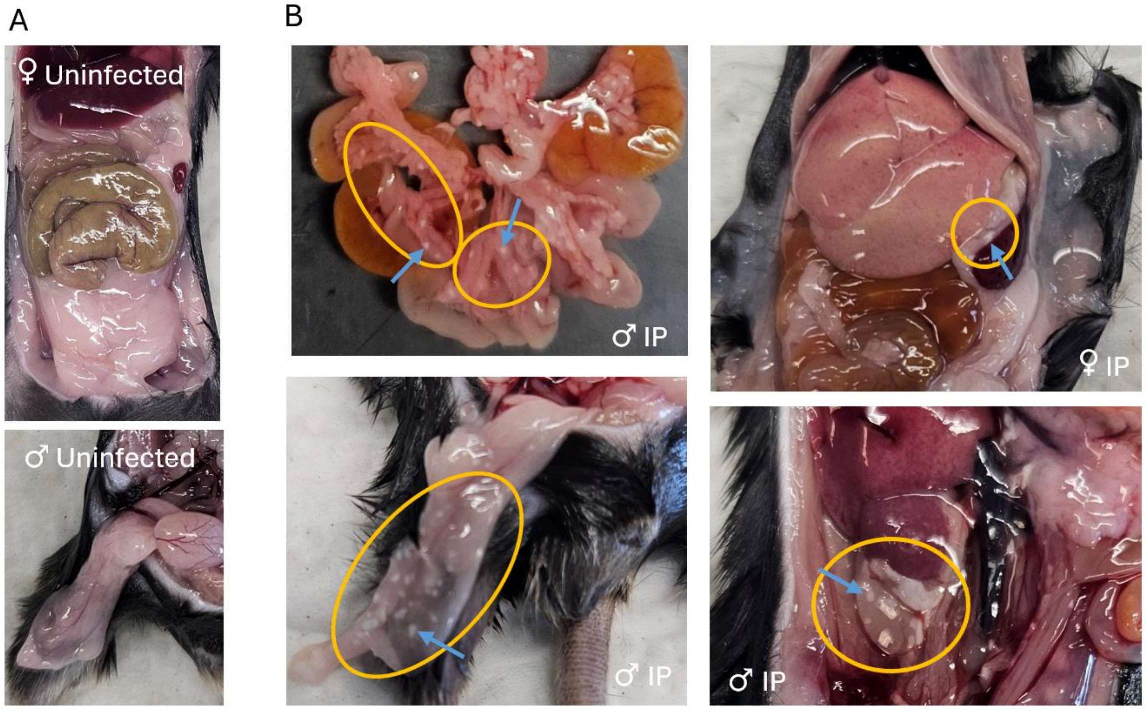

Fig. S7. Pathology observed in adipose tissue in mice. A) Images uninfected adipose. **B)** Images of white spots on mesenteric (top left), upper mesenteric (top right), perigonadal (bottom left) and perirenal (bottom right) adipose in mice. Yellow circles outline areas with pathology. Blue arrows point to focal lesions. Abbreviations: IV, intravenous; IP, intraperitoneal

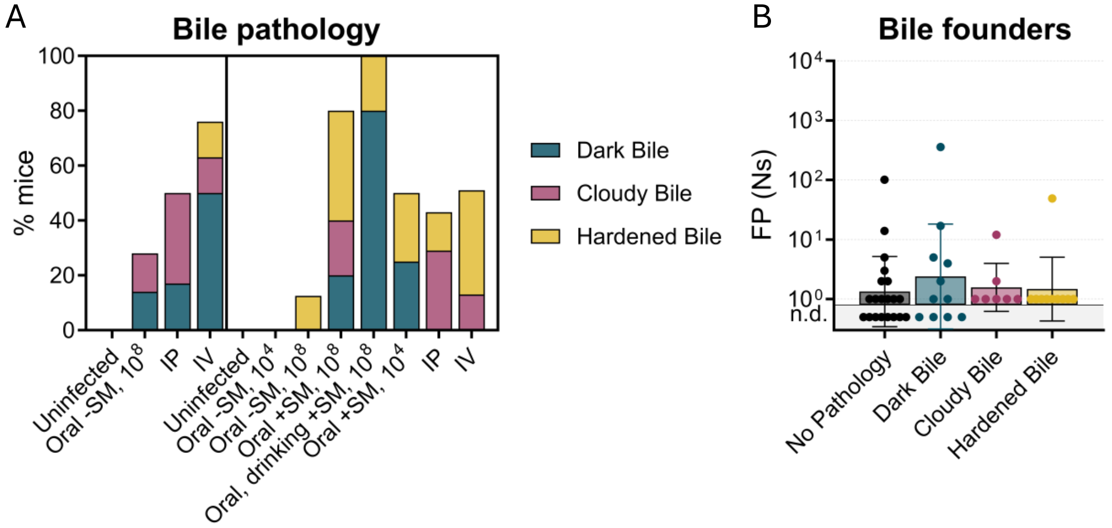

Fig. S8. Bile pathology correlates with the bile re-seeding pathway. A) Percentage of observed biliary pathology. **B)** Founding population of bile disaggregated by biliary pathology. Abbreviations: IV, intravenous; IP, intraperitoneal; SM, streptomycin; SI, small intestine

Table S1. Primer sequences for PCR.

Name	Sequence
Forward Primers	
var21	AATGATACGGCGACCACCGAGATCTACACTCTTTCCCTACACGACGCTCTTCCGATCTAATGATGGGTTAAAAAGGATCGATCC
var22	AATGATACGGCGACCACCGAGATCTACACTCTTTCCCTACACGACGCTCTTCCGATCTATGCGATGGGTTAAAAAGGATCGATCC
var23	AATGATACGGCGACCACCGAGATCTACACTCTTTCCCTACACGACGCTCTTCCGATCTTGCACGATGGGTTAAAAAGGATCGATCC
var24	AATGATACGGCGACCACCGAGATCTACACTCTTTCCCTACACGACGCTCTTCCGATCTTCATTCGATGGGTTAAAAAGGATCGATCC
var25	AATGATACGGCGACCACCGAGATCTACACTCTTTCCCTACACGACGCTCTTCCGATCTGAATCGAGATGGGTTAAAAAGGATCGATCC
var26	AATGATACGGCGACCACCGAGATCTACACTCTTTCCCTACACGACGCTCTTCCGATCTGTCAACTTGATGGGTTAAAAAGGATCGATCC
var27	AATGATACGGCGACCACCGAGATCTACACTCTTTCCCTACACGACGCTCTTCCGATCTCGGCGTGGCGATGGGTTAAAAAGGATCGATCC
var28	AATGATACGGCGACCACCGAGATCTACACTCTTTCCCTACACGACGCTCTTCCGATCTCCTGTACCTTGATGGGTTAAAAAGGATCGATCC
Reverse Primers	
AD001	CAAGCAGAAGACGGCATACGAGATCGTGATGTGACTGGAGTTCAGACGTGTGCTCTTCCGATCAGATCCTTGGCGGCAAGAAA
AD002	CAAGCAGAAGACGGCATACGAGATACATCGGTGACTGGAGTTCAGACGTGTGCTCTTCCGATCAGATCCTTGGCGGCAAGAAA
AD003	CAAGCAGAAGACGGCATACGAGATGCCTAAGTGACTGGAGTTCAGACGTGTGCTCTTCCGATCAGATCCTTGGCGGCAAGAAA
AD004	CAAGCAGAAGACGGCATACGAGATTGGTCAGTGACTGGAGTTCAGACGTGTGCTCTTCCGATCAGATCCTTGGCGGCAAGAAA
AD005	CAAGCAGAAGACGGCATACGAGATCACTGTGTGACTGGAGTTCAGACGTGTGCTCTTCCGATCAGATCCTTGGCGGCAAGAAA
AD006	CAAGCAGAAGACGGCATACGAGATATTGGCGTGACTGGAGTTCAGACGTGTGCTCTTCCGATCAGATCCTTGGCGGCAAGAAA
AD007	CAAGCAGAAGACGGCATACGAGATGATCTGGTGACTGGAGTTCAGACGTGTGCTCTTCCGATCAGATCCTTGGCGGCAAGAAA
AD008	CAAGCAGAAGACGGCATACGAGATTCAAGTGTGACTGGAGTTCAGACGTGTGCTCTTCCGATCAGATCCTTGGCGGCAAGAAA
AD009	CAAGCAGAAGACGGCATACGAGATCTGATCGTGACTGGAGTTCAGACGTGTGCTCTTCCGATCAGATCCTTGGCGGCAAGAAA
AD010	CAAGCAGAAGACGGCATACGAGATAAGCTAGTGACTGGAGTTCAGACGTGTGCTCTTCCGATCAGATCCTTGGCGGCAAGAAA
AD011	CAAGCAGAAGACGGCATACGAGATGTAGCCGTGACTGGAGTTCAGACGTGTGCTCTTCCGATCAGATCCTTGGCGGCAAGAAA
AD012	CAAGCAGAAGACGGCATACGAGATTACAAGGTGACTGGAGTTCAGACGTGTGCTCTTCCGATCAGATCCTTGGCGGCAAGAAA
AD013	CAAGCAGAAGACGGCATACGAGATTTGACTGTGACTGGAGTTCAGACGTGTGCTCTTCCGATCAGATCCTTGGCGGCAAGAAA
AD014	CAAGCAGAAGACGGCATACGAGATGGAACTGTGACTGGAGTTCAGACGTGTGCTCTTCCGATCAGATCCTTGGCGGCAAGAAA
AD015	CAAGCAGAAGACGGCATACGAGATTGACATGTGACTGGAGTTCAGACGTGTGCTCTTCCGATCAGATCCTTGGCGGCAAGAAA
AD016	CAAGCAGAAGACGGCATACGAGATGGACGGGTGACTGGAGTTCAGACGTGTGCTCTTCCGATCAGATCCTTGGCGGCAAGAAA
AD018	CAAGCAGAAGACGGCATACGAGATGCGGACGTGACTGGAGTTCAGACGTGTGCTCTTCCGATCAGATCCTTGGCGGCAAGAAA

AD019	CAAGCAGAAGACGGCATACGAGATTTTCACGTGACTGGAGTTCAGACGTGTGCTCTTCCGATCAGATCCTTGGCGGCAAGAAA
AD020	CAAGCAGAAGACGGCATACGAGATGGCCACGTGACTGGAGTTCAGACGTGTGCTCTTCCGATCAGATCCTTGGCGGCAAGAAA
AD021	CAAGCAGAAGACGGCATACGAGATCGAAACGTGACTGGAGTTCAGACGTGTGCTCTTCCGATCAGATCCTTGGCGGCAAGAAA
AD022	CAAGCAGAAGACGGCATACGAGATCGTACGGTGACTGGAGTTCAGACGTGTGCTCTTCCGATCAGATCCTTGGCGGCAAGAAA
AD023	CAAGCAGAAGACGGCATACGAGATCCACTCGTGACTGGAGTTCAGACGTGTGCTCTTCCGATCAGATCCTTGGCGGCAAGAAA
AD025	CAAGCAGAAGACGGCATACGAGATATCAGTGTGACTGGAGTTCAGACGTGTGCTCTTCCGATCAGATCCTTGGCGGCAAGAAA
AD027	CAAGCAGAAGACGGCATACGAGATAGGAATGTGACTGGAGTTCAGACGTGTGCTCTTCCGATCAGATCCTTGGCGGCAAGAAA

Dataset S1 (separate file). Barcode counts for all samples in manuscript.
